# A Systematic Review of Attributes Influencing Preferences for Treatments and Interventions in People With Amyotrophic Lateral Sclerosis (ALS)

**DOI:** 10.1002/mus.28437

**Published:** 2025-05-23

**Authors:** A. Clift, D. Rowen, L. Knox, A. W. Griffiths, C. J. McDermott

**Affiliations:** ^1^ Sheffield Institute of Translational Neuroscience University of Sheffield, Sheffield Institute of Translational Neuroscience, University of Sheffield Sheffield UK; ^2^ Sheffield Centre for Health and Related Research, University of Sheffield, Sheffield Centre for Health and Related Research University of Sheffield Sheffield UK; ^3^ Academic Directorate of Neuroscience, Sheffield Teaching Hospital NHS Foundation Trust, Academic Directorate of Neuroscience Sheffield Teaching Hospital NHS Foundation Trust Sheffield UK

**Keywords:** ALS, interventions, MND, preferences, treatments, views

## Abstract

Amyotrophic lateral sclerosis (ALS) is a progressive neurodegenerative disease that has no cure, and treatments predominantly focus on improving quality of life. Patient‐centred care is central to bringing about meaningful improvements to quality of life. This review addresses the lack of consolidated evidence on what matters most to people with ALS (pwALS) by synthesizing 44 preference‐based studies covering six different treatment and intervention categories. Data‐based convergent synthesis identified five overarching factors influencing preferences: ease of use, accessibility, making life easier, autonomy, and safety/reliability. Simplifying and enhancing accessibility of treatment delivery across disease stages aligns with the nature of neurodegenerative disorders such as ALS, where function declines as the disease progresses. The value in perceived and real control reflects the profound impact ALS has on an individual's independence. Safety and reliability are crucial for people with ALS and are recognized as fundamental requirements for quality healthcare. The themes identified in this review can inform the attributes of preference elicitation methods. Systematically varying the levels of these attributes elicits quantitative measures of preferences. These findings can be used to inform and develop healthcare policy and clinical practice in ALS care. Specifically, preferences related to drug treatments can then be integrated into target product profiles (TPPs) to align drug development with the needs and values of pwALS. Integrating patient preferences into clinical practice promotes patient‐centred care, increasing both patient satisfaction and treatment effectiveness.

**Abbreviations**
ALS: amyotrophic lateral sclerosis
HCPs: Health care professionals
IDDD: Implanted drug delivery device
NIV: non‐invasive ventilation,
PEG: percutaneous endoscopic gastrostomy
PD‐TURSO: sodium phenylbutyrate and taurursodiol
pwALS: people with ALS
THC: CBD tetrahydrocannabinol and cannabidiol

## 1 Introduction

Understanding the preferences of patients towards treatments and interventions goes beyond assessing effectiveness; it evaluates the acceptability and desirability, revealing the underlying factors influencing adherence. Such insights can be used to adjust and tailor care to the wishes of the patient [1, 2]. Adopting this approach has consistently been shown to increase treatment satisfaction and adherence, and ultimately improve patients' quality of life [3].

In many conditions without a cure, the focus of care is improving quality of life. Patient‐centered care allows healthcare interventions to consider individual needs, bringing about meaningful improvements to life [4, 5]. The treatments for amyotrophic lateral sclerosis (ALS) encompass both disease‐modifying and symptomatic approaches [6]. Disease‐modifying drugs vary in efficacy, administration, side effects, and cost [7]. Global regulatory differences further complicate treatment availability [8]. As disease‐modifying treatments offer only modestly effective benefits in altering the disease's progression, optimizing quality of life remains central to ALS care [9, 10]. Beyond drug therapies, nutritional support, psychosocial care, physiotherapy, and assistive equipment (including communication aids and respiratory support) are crucial for managing symptoms and enhancing quality of life [11]. Digital health tools are also increasingly used to improve care accessibility and remote monitoring of the disease [12, 13].

It is particularly important to measure preferences in ALS as treatment and intervention options are complex and may require trade‐offs between quality of life and treatment burden [14]. As more treatments and interventions are developed, it becomes increasingly important to ensure the preferences of people with ALS (pwALS) are taken into account [15]. This review synthesizes existing evidence around what is important to pwALS for ALS treatments and interventions, and their characteristics.

## 2 Materials and Methods

### 2.1 Information Sources and Search Strategy

The study protocol was registered on PROSPERO [CRD42024526017].

The search strategy used free‐text and thesaurus search terms for:

(i) Motor Neuron(e) Disease and Amyotrophic Lateral Sclerosis (ii) Preferences, willingness to pay, attitudes towards, decision making, experiences, expectations, satisfaction, perceptions, perspectives (iii) Drug, medication, treatment, intervention, disease modification, symptom management, service, healthcare.

A single search was conducted in PubMed, Scopus, CINAHL and PsychINFO in which search terms (i), (ii) and (ii) were combined using “AND” to identify articles regarding preferences in people with ALS. Backward citation searching was also conducted. Initial searches were carried out in April 2024 and re‐run in September 2024. An information specialist supported the development of the search strategy and choice of databases.

Due to international inconsistency in terminology, searches have been conducted for both the terms motor neuron disease (MND) and amyotrophic lateral sclerosis (ALS). Searches were conducted from 2011 onwards to build on a previous review [16] and systematically search current literature that has not yet been synthesized. Full inclusion and exclusion criteria can be found in the Supporting Information (Table A1).

### 2.2 Data Collection Process

All references obtained from the various databases were uploaded and duplicates removed [17]. One author (AC) conducted a two‐stage screening process. First, titles and abstracts were assessed against the established inclusion/exclusion criteria. Second, the eligibility of relevant studies was determined by reading the full manuscripts. Any uncertainties were resolved by discussion among the authors. Rayyan was used as an online organizational tool, facilitating the sorting of citations. The stages of screening are reported in alignment with the Preferred Reporting Items for Systematic Reviews and Meta‐Analyses (PRISMA 2020) statement [18]. Studies that met all aspects of full text screening were included for review for data extraction, synthesis, and critical review.

### 2.3 Data Extraction

Data extraction follows a framework and extracted data is displayed in tabular forms focusing on: (1) Publication details (2) Type of study (3) Study sample (4) Study characteristics and (5) study outcomes.

### 2.4 Data Synthesis

A mixed methods synthesis was conducted to integrate the findings from all the studies. Reflexive thematic analysis was conducted systematically and iteratively on the qualitative data [19]. Line‐by‐line open coding was conducted on the extracted data, identifying initial codes related to patient preferences (AC). The codes were then reviewed and grouped into broader categories, looking for patterns and relationships between them (all authors). This process involved multiple rounds of discussion and refinement, with each reviewer independently analyzing the coded data and then comparing their interpretations. Discrepancies were resolved through discussion until consensus was reached. Numerical quantitative data was extracted and then synthesized using a narrative summary approach due to the heterogeneity across studies in terms of outcome measures and study designs [20]. Where possible, numerical data on preferences were presented to highlight trends. Both qualitative and quantitative data from mixed‐methods studies were fully extracted using one of the above processes to ensure a comprehensive understanding of the preferences [21]. The integration of qualitative and quantitative findings was conducted using a data‐based convergent synthesis approach [22].

### 2.5 Quality Assessment

The quality of each study was assessed by AC using the Mixed Method Appraisal Tool (MMAT) [23]. The MMAT was used to assess the methodological quality of included studies, evaluating their research questions, data collection, analysis methods, no response bias (for quantitative studies) or rationale for the mixed methods approach (for mixed methods studies). A second reviewer (AWG) assessed 15.5% of the studies for inter‐rater reliability and to discuss any discrepancies. A sensitivity analysis was then performed.

## 3 Results

**FIGURE 1:**
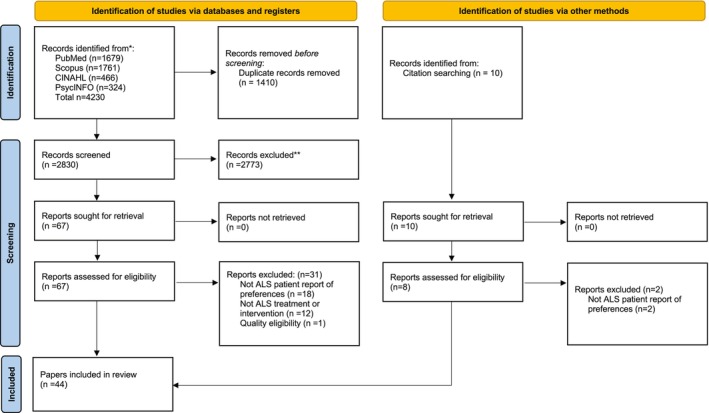
PRISMA showing the flow of papers during screening for the systematic review on the preferences of people with amyotrophic lateral sclerosis (ALS) towards ALS interventions.

A total of 4240 papers were identified in searches across 4 databases and through backward citation searching. Of these, 77 papers were included after title/abstract screening, and 44 papers were included in the review (See Figure 1).

A well‐defined categorization of treatments and interventions in ALS includes drug treatments, nutrition, special equipment, psychosocial support, physiotherapy, exercise programmes, and mobility aids [11]. This framework was used to classify studies and, within each category, subcategories were created to further explore findings. This resulted in the following: (1) drug treatments (symptom management and disease modifying) (2) nutrition, (3) special equipment, (4) psychosocial support, (5) exercise programmes. Additionally, a sixth intervention type was introduced: (6) digital health, encompassing telehealth, telecare, and telemonitoring.

**FIGURE 2:**
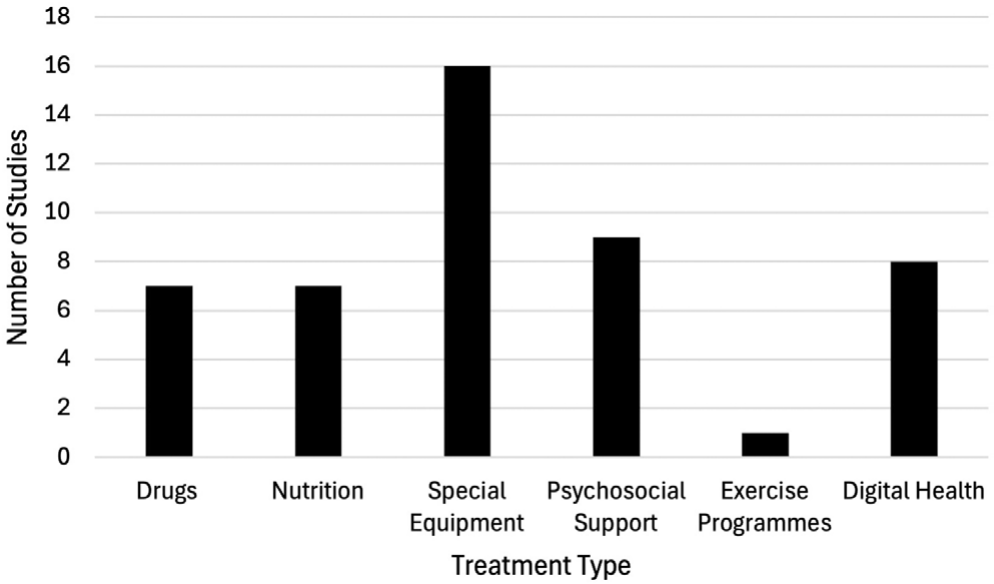
Number of articles published per category of intervention.

The number of articles published per treatment type shows the highest number of studies were of specialist equipment (*n* = 16). Two papers report on both nutrition (percutaneous endoscopic gastrostomy (PEG)) and respiration (non‐invasive ventilation (NIV)) studies within a single paper [24, 25] (See Figure 2). There were 19 quantitative studies, 15 qualitative studies, and 10 mixed methods studies. Studies were conducted across seven countries in Europe (*n* = 25), two countries in North America (*n* = 9), across two countries in Australasia (*n* = 1) and in South America (*n* = 1) and Asia (*n* = 1). Some studies were conducted across multiple locations. Details regarding the characteristics of the studies can be found in the Supporting Information (Table A2).

Eight quantitative studies did not achieve a representative sample of the target MND population, due to a small sample, only recruiting from one ALS clinic and/or only including participants who had accepted/refused a treatmentt [25, 26, 27, 28, 29, 30, 31, 32]. There are concerns about generalization to a wider MND population due to the limited diversity in characteristics of participants. Nine studies had unjustified low response rates, risking non‐response bias due to potential systematic differences between participants and non‐participants [27, 28, 30, 31, 32, 33, 34, 35, 36]. For more details and the complete MMAT see Supporting Information (Table A3).

**FIGURE 3:**
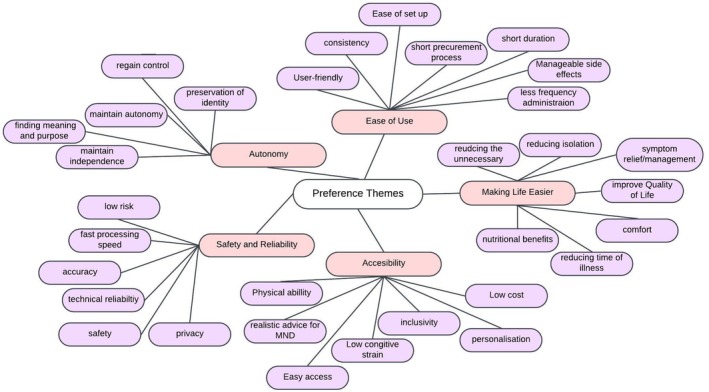
Thematic analysis map of the identified preferences across treatment categories.

The results are presented by treatment/intervention categories: drugs, nutrition, special equipment, psychosocial support, exercise programmes, and digital health. Findings from the review are described under these categories using the themes/subthemes extracted from thematic analysis (See Figure 3). Not all themes/subthemes apply to each treatment/intervention as their relevance reflects the treatment/intervention goals.

### 3.1 Drugs

#### 3.1.1 Ease of Use

Most pwALS recruited from four European countries faced challenges in swallowing riluzole that led to treatment delays or omissions (Table 1). These people had the strongest preference for a new formulation with an easier mode of delivery that dissolved quickly on the tongue and had convenient/portable packaging [37]. Similarly, an implanted drug delivery device (IDDD) was considered to be a preferred alternative to a lumbar puncture for intrathecal therapy by pwALS in multiple clinical settings in the US and Europe, due to it being perceived as an easier mode of delivery [38]. PwALS valued a shorter duration and less frequent administration of the treatment [38]. Despite a general acceptance of edaravone, 22/331 (6.6%) participants voluntarily suspended this potential disease‐modifying drug treatment due to the burden of the intravenous route of administration [33].

**TABLE 1:** Summary of preferences results for drug treatment studies.

Author	Preference assessment	First order participant quotes/primary data from the studies.	Second order codes	Preference theme
Ludolf et al. [37]	Patient Preference Survey (PPS)	Low risk of choking and underdosing. Easier mode of delivery	Low risk Ease of administration	Safety/Reliability Ease of Use
Seo et al. [38]	Discrete choice experiment	The risk of device failure. Shorter overall durations and less frequent administration	Low risk Less disruptive administration	Safety/Reliability Ease of Use
Lunette et al. [33]	Observational assessment	22 patients voluntarily suspended from the burden of the duration and route of administration.	Shorter duration Easier route of administration	Ease of Use
Zubair et al. [39]	Semi structured Interviews	“It would be good to have a standard syringe, because otherwise, you have to learn every time.” “The cost poses a barrier	Easier administration Cost	Ease of Use Accessibility
Quinn et al. [34]	Patient report recorded by carers	The main reasons for not taking were discomfort from the gastrointestinal side effects 17/29 (58.6%) and from the taste of the drug 8/29 (29.6%).	Side effects Taste	Ease of Use
Meyer et al. [40]	Net Promoter Score (NPS), Treatment satisfaction questionnaire (TSQM‐9)	10/32 found it difficult to use. 5/32 found it inconvenient or very inconvenient to use.	Ease of Use	Ease of Use
Jia et al. [41]	Questionnaire then Interviews	“at least it doesn't have so many side effects” “Riluzole is too expensive. If it is cheaper, I would try it”	Side effects Cost	Ease of use Accessibility

Difficulties were expressed with the range of syringes available for the injection of methylcobalamin, a form of vitamin B12 with limited evidence of potential slowing of functional decline in ALS. It was reported “It would be good to have a standard syringe, because otherwise, you have to learn every time” [39]. A single ALS centre observed the use of PB‐TURSO, a combination of sodium phenylbutyrate (PB) and taurursodiol (TURSO) for the disease modification of ALS. The study observed a high discontinuation rate due to gastrointestinal side effects 17/29 (58.6%) and the drug's taste 8/29 (27.6%) [34].

The ease of administration is also important to pwALS when receiving drug treatments to manage the symptoms of their ALS. Almost all people with ALS were satisfied with the effectiveness of tetrahydrocannabinol and cannabidiol (THC:CBD) for treating symptoms of spasticity, but a third reported issues with the ease of administration of the oromucosal spray, highlighting the way this restricts usability [40].

#### 3.1.2 Accessibility

PwALS express the importance of affordability in deciding whether to start or continue a drug treatment. “*Riluzole is too expensive. If it is cheaper, I would try it*” [41]. Similar concerns were expressed regarding vitamin B12 injections, where “*The cost poses a barrier to how often I take the [vitamin B12] shots now.29*”.

#### 3.1.3 Safety/Reliability

Almost all pwALS reported a low risk of choking as the leading factor when choosing a drug formulation [37]. For the intrathecal delivery of drugs, pwALS valued a low risk from the drug administration and low frequency of administration. There was a preference for an IDDD compared to a lumbar puncture, although participants in this survey were only willing to accept a low risk of device failure to be able to switch from lumbar puncture to an IDDD [38].

### 3.2 Nutrition

#### 3.2.1 Autonomy

Five studies found the loss of control and social enjoyment at mealtimes formed barriers to accepting PEG [24, 25, 42, 43, 44] (Table 2). One participant “wanted to continue to eat independently, no matter how difficult it was.” [25].

**TABLE 2:** Summary of preference results for the nutrition studies.

Author	Preference assessment	First order participant quotes/primary data from the studies.	Second order codes	Preference theme
Labra et al. [42]	Interviews and analysis of physical function	“I am thinking that it will help me because you can still go out” “I can use PEG to keep up my nutrition”	Maintaining independence Nutritional benefit	Autonomy # Making life easier
Guillemin et al. [45]	Survey	“It is difficult to use knives and other utensils” 6/10 (60%) had weight loss concerns.	Ease in food preparation Nutritional benefit	Making life easier
Starvoulakis et al. [43]	Semi structured interviews 3 months post PEG	“Swallowing was becoming more difficult, “That's when she said, ‘Let's get it done”.	Prolonged, tiring and effortful meals Choking and aspiration	Making life easier
van Eenennaam et al. [44]	Semi structured Interviews	“I also wanted to continue to eat independently, no matter how difficult it was”.	Feeling of control	Autonomy
Hogden, Labra and Power [46]	Semi structured interviews	“My main reason for getting the PEG was my swallowing”	Optimizing quality of life	Making life easier

#### 3.2.2 Making Life Easier

When food preparation [45] and eating [42, 43] became challenging and less enjoyable, individuals were more inclined to accept PEG to simplify life. One participant explained, *“My main reason for getting the PEG was my swallowing*,” highlighting issues with choking and aspiration as key factors in the decision, along with the belief that it would improve quality of life [46].

### 3.3 Special Equipment

#### 3.3.1 Ease of Use

When receiving non‐invasive ventilation (NIV) the sensation of air being blown into the mask at high pressure was described as *“too powerful”* and difficulties were reported with wearing glasses or maintaining physical closeness with partners [47] (Table 3). Participants highlighted challenges with mask adjustment, particularly with clips, stating, “*I can get the mask on… but I can't, it's the clips*” [47]. In contrast, the simplicity of using a cough assist was positively anticipated: “It is not very complicated either. I think that will be important” [26].

**TABLE 3:** Summary of preference results for the special equipment studies.

Author	Preference assessment	First order participant quotes/primary data from the studies	Second order codes	Preference theme
Greenaway et al. [24]	Semi structured interviews	“As far as I'm concerned it's my life, what's left” “Yeah, it's the worry that it'll get infected”	Perceptions of choice and control Aspects of fear	Autonomy Safety and Reliability
Martin et al. [25]	Interviews and beliefs about medicine questionnaire (BMQ)	More pleasure in eating were more likely to have refused an intervention	Control over illness	Autonomy
Ando et al. [48]	Semi structured interviews	“I wouldn't want it to be all of the time and not have any life” “claustrophobia – I just don't want it.”	Preservation of the self Negative perceptions of mask	Autonomy Safety and Reliability
Baxter et al. [47]	Semi structured interviews in first month of NIV	“I can get the mask on…but I can't, it's the clips.” “leaks just from the mask” “that mask on my face”	Ease of set up Mask dysfunction Negative perceptions of the mask.	Ease of Use Safety and Reliability
Siewers, Holmoy & Frich, [26]	Interviews	“it's not very complicated either. I think that will be important”. “So knowing that it is there	Ease of Use Comfort in reliability	Ease of Use Safety and Reliability
Huggins, Wren & Gruis, [27]	Survey using 1–10 scale	Accuracy followed by simplicity as most important. Appearance was least important	Accuracy	Safety and Reliability
Geronimo et al. [49]	10 min demonstration then survey	38/42 (90.5%) of ppts would like 90% accuracy and 50% required at least 80%.	Accuracy	Safety and Reliability
Eicher et al. [50]	Semi structured interviews	“It would be great if could also disburden somebody” patient was afraid…it might fall off the ceiling and hit him	Maintain and retrieve autonomy Safety	Autonomy Safety and Reliability
Creemers et al. [51]	Survey	Long duration of application process, paperwork, knowledge and errors by supplier	Delays in procurement process	Ease of Use
Riera‐Punet et al. [28]	Intervention for 3 months then questionnaire	The main reason for not using it was discomfort. Difficulty making an impression to make the mold due to MND	Comfort Physical barriers	Ease of Use Accessibility
Spears et al. [35]	Online Survey	“Too uncomfortable” (51%) and “too restricting” (44%) led to users not wearing. (96%) want the collar to be well fitting.	Comfort	Ease of Use
Mackenzie et al. [52]	Survey with closed and open questions	“Since my hands ceased being able to move, I have been isolated from this”. “MND stops a person being independent, but with a computer (she) was able to communicate”.	Lack of physical ability Reduce isolation	Accessibility Making life easier
Peters, O'Brien & Fried‐Oken, [29]	Questionnaire	high cost due to insurance co‐payments. Concerns about using the device and it not working well	Cost Risk of device failure	Accessibility Safety and Reliability
Caligari et al. [53]	Quebec User Evaluation of Satisfaction with Assistive Technology (QUEST 2.0)	Side effects of bloodshot eyes from use. Regaining autonomy in personal life choices	Side effects Regained autonomy	Ease of Use Autonomy
Cave and Bloch, [54]	Interviews	“The voice helps me retain something of me.” “It's about control”	Preserving identity Control	Autonomy
Spittel et al. [30]	User experience survey	Issues included the unsuitability of the device 16/53 (30.2%) followed by rejection by the health care insurance 15/53 (28.3%)	Adaptability to MND Cost	Accessibility

Keeping hospital admission to a minimum was important when considering a brain computer interface, as 44/61 (72%) would accept surgically implanted electrodes with outpatient surgery, but willingness decreased to 25/61 (41%) if it involved a hospital stay [27]. This finding underscores a strong preference for procedures that are minimally disruptive to day‐toto‐day life.

#### 3.3.2 Accessibility

The lack of adaptability of many assistive technologies to the specific needs of ALS was a barrier to use [50]. 89/179 (49.7%) pwALS report the application process for assistive technology and home adaptations as a key barrier [51]. Among users of augmentative and alternative communication (AAC) devices, high insurance co‐payments prevented 5/174 (2.9%) of pwALS from using these devices [29].

#### 3.3.3 Autonomy

Many pwALS expressed a preference for low dependence on non‐invasive ventilation (NIV). One participant explained “I wouldn't want it to be all of the time and not have any life” [48]. However, as this sample included only participants who had declined NIV, these preferences may reflect unique perspectives on autonomy among this group. Assistive home devices were valued due to their ability to maintain and retrieve autonomy as “it would be great if it could also disburden somebody” and allow for more independence [50]. Voice banking was a preferred means of preserving personal identity and retaining autonomy in personal life choices [53, 54]. Additionally, communication aids were favored for their role in reducing isolation and supporting independent communication [52].

#### 3.3.4 Safety/Reliability

PwALS expressed a preference for NIV masks that function reliably: “It leaks just from the mask, it's not very good” [47]. Some participants said that the enclosed design of the mask could feel restrictive and potentially unsafe [48]. Cough assist devices were particularly valued for their reliability with one participant expressing “knowing that it is there” provided a sense of security for unpredictable needs [26]. PwALS desired eye‐tracking technology and brain‐computer interfaces (BCIs) to be both accurate and reliable [27, 29, 49]. One BCI paper showed 21/42 (50%) participants required there to be at least 80% accuracy while 38/42 (90.5%) would like the device to have 90% or higher accuracy [49]. PwALS showed little concern for the appearance of these devices [27]. There were safety concerns for home assistive devices, particularly regarding secure installation with one participant fearing that a ceiling‐mounted system might fall, and they would be unable to move to safety due to their ALS [50].

### 3.4 Psychosocial Support

#### 3.4.1 Ease of Use

PwALS expressed a desire for continuity in care providers in respite care, emphasizing that frequent transitions required constant “readjusting and [caregivers] re‐learning,” which detract from the ease and enjoyment of the intervention [55] (Table 4).

**TABLE 4:** Summary of preference results for the psychosocial support studies.

Author	Preference assessment	First order participant quotes/primary data from the studies.	Second order codes	Preference theme
Weeks et al. [56]	Individual semi structured interviews then discussion groups	“Having a therapist coming home would be very useful” “can't physically communicate	Adaptability to MND.	Accessibility
Pinto et al. [57]	Think aloud interviews then intervention for 6 weeks then in‐depth interviews.	More appropriate I think to people with motor neuron” Taking some control over what is happening to them.	Realistic advice for MND Regaining control	Accessibility Autonomy
Hardy, Castle & Jackson, [31]	Survey with 5‐point Likert scale	Value the availability of psychiatric services even when have no symptoms	Accessibility	Accessibility
Marconi et al. [58]	Weekly meditation for 8 weeks followed by interviews	It was difficult for us to organize transportation” “wasn't easy to do exercises at home”	Transportation Continuity	Accessibility
Bentley et al. [32]	Dignity therapy followed by feedback questionnaire	Positive improvements in continuity of self and role preservation.	Preservation of identity	Autonomy
Sommers‐Spijkerman et al. [59]	Survey and Interviews	“Thanks to this app I think ‘What positive things happened today?’” Participants struggled to integrate the intervention into everyday life 10/13 (63%).	Positive shift in attention Continuity	Making life easier Accessibility
Wu et al. [55]	Interviews	Readjusting and them re‐learning” “wouldn't want them to bathe her”	Consistency Privacy	Ease of Use Autonomy

#### 3.4.2 Accessibility

Accessibility is a key preference in psychosocial support particularly inclusivity of the varied needs associated with ALS such as communication difficulties [56, 57]. Participants suggested practical improvements such as “having a therapist coming home would be very useful” and “*text reminders, and written information”* [56]. Ensuring the continued availability of psychological interventions at home following in‐person services is important as many participants struggled to integrate these interventions into their daily lives [59], with one noting, “At the beginning, it wasn't easy to do exercises at home exactly the way trainers taught us” [58].

#### 3.4.3 Autonomy

Preserving independence and maintaining privacy was highly valued by individuals with ALS. This has been shown to exempt close families, with one participant explaining during respite care “I have trouble asking other people besides my husband to do things for me” [55]. Dignity therapy was positively received for its support of autonomy and found positive reports from the continuity of self, acceptance and role preservation [32].

### 3.5 Exercise Programmes

#### 3.5.1 Making Life Easier

PwALS value exercise programs that enhance daily life such as through a sense of achievement, reduced immobility, and improved well‐being. Symptom relief was also significant, with reduced limb rigidity and muscle stiffness, along with improvements in flexibility, muscle preservation, sleep quality, and strength as key factors [60] (Table 5).

**TABLE 5:** Summary of preference results for the exercise programme studies.

Author	Preference assessment	First order participant quotes/primary data from the studies.	Second order codes	Preference theme
Maier et al. [60]	5‐ and 11‐point Likert scale, Net Promoter Score (NPS) and interviews	Key impacts include a sense of achievement (67%), reduced immobility (61%), and improved well‐being (55%). Symptom relief with reduced limb rigidity (63%) and muscle stiffness (52%).	Improved quality of life Symptom relief	Making life easier

### 3.6 Digital Health Tools

#### 3.6.1 Ease of Use

PwALS expressed a willingness to use teleconsultations and home monitoring but wanted these digital health tools to be straightforward and easy to operate [6, 36, 61] (Table 6).

**TABLE 6:** Summary of preference results for the digital health tool studies.

Author	Preference assessment	First order participant quotes/primary data from the studies.	Second order codes	Preference theme
Fidelix et al. [36]	Teleconsultation followed by questionnaire	4/36 (11%) patients disagreed that it was easy to set up The convenience of receiving assistance and maintenance of multidisciplinary care at home.	Ease of set up Continuity of care	Ease of Use Accessibility
Helleman et al. [62]	Survey then interviews	““Logging in is difficult with the digital ID” “You don't feel you are going for no good reason” Positive about receiving personalized feedback and information	Log in issues Reducing the unnecessary Personalisation	Ease of Use Making life easier Accessibility
James et al. [63]	Survey then interviews	“It wouldn't take so much energy out of my life and I think my day would be a bit better”	Reducing the unnecessary	Making life easier
Hobson et al. [64]	Questionnaire then interviews	“Anything that makes life's journey, when necessary, better” 9/12 said they would use technology with the appropriate equipment and training.	Making life easier Accessibility	Making life easier Accessibility
Ando et al. [65]	Telemonitoring for NIV for 24 weeks then interviews	“It saves a lot of money as well as you know because I won't need to keep going to hospital” “The keyboard is that frustrating. I just couldn't be bothered trying to get it to work because it wouldn't.”	Reducing the unnecessary Technical challenges	Making life easier Safety and reliability
Helleman et al. [66]	Survey	86.1% were willing to record at least monthly and 60.7% at least weekly. Concerns with privacy, data security and data being sold to third parties.	Low cognitive burden Technical reliability	Accessibility Safety and reliability
Tattersall et al. [67]	At home assessments via webcam then a survey	Difficulty from mobility and speech issues Reducing the number of clinic visits was valuable in 24/25 of the pwALS	Adaptability to MND Reducing appointments	Accessibility Making life easier
Beswick et al. [61]	Questionnaire	Difficulty putting on the devices and faulty straps. Large size of the device affected their comfort Positive response that it meant less clinical appointments.	Physical ability, Comfort Reducing appointments	Ease of use Accessibility Making life easier

#### 3.6.2 Accessibility

PwALS appreciated having continued access to telehealth interventions as “*If you've got a problem, no matter what time it is, you can type it in.”* [65]. Simplified interfaces were preferred, as frustration and low usage were linked to difficulties using the messaging system and on‐screen keyboard of a telecommunication device, primarily due to reduced fine motor skills [64, 65]. Most participants reported a willingness to use the technology if it had appropriate adaptive equipment [64]. Additionally, 277/322 (86.1%) of participants were willing to record data at least monthly, with 195/322 (60.7%) willing to do so weekly, emphasizing the need for low‐burden tools that accommodate cognitive demands [66].

#### 3.6.3 Making Life Easier

PwALS positively perceive digital health tools including videoconferencing and telemonitoring of NIV that reduces in person clinical appointments and thus avoids unnecessary costs [66]. This was a *“life saver”* [64] and participants found the convenience of digital tools beneficial for everyday life [61, 63, 64, 65, 67]. However, while these tools were appreciated, it was also acknowledged that pwALS may not want to become “trapped in the house” highlighting the importance of balancing remote care with opportunities for social engagement and mobility [63]. It was suggested to have initial face‐to‐face contact with the ALS clinic that is later changed to being delivered remotely.

#### 3.6.4 Safety and Reliability

Although generally positive about movement‐evaluating devices, some people with ALS experienced charging problems that hindered use [61]. A preference for trustworthy technology was indicated as pwALS expressed concerns about privacy, data security, and the potential sale of personal data [66].

## 4 Discussion

Across six treatment and intervention categories, five overarching factors influenced preferences: ease of use, accessibility, making life easier, autonomy, and safety/reliability. Not all treatments addressed every theme or subtheme, likely reflecting the number of studies or the priorities of pwALS.

Ease of use is key for drug treatments in which the method and frequency of delivery were of greatest importance. PwALS showed an openness towards exploring and accepting new drugs and drug delivery methods even when effectiveness is unclear [37, 38, 40]. Similar findings are observed in Huntington's Disease (HD) and spinocerebellar ataxia (SCA) where the mode and frequency of administration significantly influenced preferences. For instance, respondents favored a single operation over repeated lumbar punctures [68] as was reported for the intrathecal delivery of ALS drugs [38]. There is increasing recognition of the value in involving views of people living with a disease or condition throughout the decision making of drug treatments development lifecycle [69]. Building on the attributes of ease of use, accessibility, and safety/reliability identified in this review, specific to drug studies, quantitative methods of preference elicitation, such as discrete choice experiments (DCEs), can be conducted. These methods can elicit preference data regarding specific levels of the attributes—for example, the preferred frequency of drug administration. These preferences can then be integrated into target product profiles (TPPs) to align drug development with the needs and values of pwALS.

The preference for ease of use extends beyond drug treatment to interventions such as communication aids [29, 52] and is closely linked to accessibility, both physically and cognitively. Accessibility is particularly important for pwALS and other progressive neurodegenerative diseases in whom mobility and function can rapidly decline and make traditional care difficult to implement [70]. Prioritizing both ease of use and accessibility ensures effective and continued care throughout disease progression.

Safety and reliability are important factors for pwALS and are recognized as fundamental requirements for quality healthcare [71]. The preference for safety is observed in multiple sclerosis (MS) clinics where patients taking injectable treatments placed the most concern on the risk of serious infection [72]. In contrast, a study investigating preferences for methods of delivering disease‐modifying drugs for HD and SCA found associated risks did not influence the preference for intrathecal drug delivery [68]. This suggests safety and reliability do not universally affect decision making in all neurodegenerative diseases and may be particularly important in ALS due to the fast progression and increased vulnerability of individuals. Reliable interventions are therefore critical to ensuring continuity of care and minimizing disruptions that could threaten life, independence, and increase caregiver burden.

This review builds on a previous systematic review on perceptions and preferences to services and care in ALS [16]. Both reviews highlight a consistent emphasis on the importance of continuity and accessibility in care, alongside interventions designed to preserve autonomy and support independence. These priorities have been particularly evident over time in the use of augmented and assisted communication (AAC) aids and home adaptations to facilitate daily living. A key distinction of this review is the broader range of treatment categories, including the preferences of pwALS for drug treatments, particularly disease‐modifying drugs. Available or experimental disease‐modifying drugs are constantly evolving, and it is important to understand patients' preferences and incorporate them into the development process. Collating these preferences will have significant implications for policy making and drug development and aid in the alignment of treatments with the needs and expectations of those living with ALS. This review also examines digital health tools and advanced methods like brain‐computer interfaces, highlighting the importance of technological reliability and accuracy in these novel interventions [27, 49, 61, 62]. The previous review shows most studies emanating from Europe and North America [16]. Whilst this remains true, underscoring a continued overreliance on the views of white, western populations, this review includes more global distribution, with research from Europe, North America, Australasia, and some studies from South America and Asia. This wider geographic scope suggests a broader effort to understand ALS treatment preferences across diverse populations.

Different healthcare systems offer varying levels of access to diagnosis, treatment, supportive care, and end‐of‐life services for people with ALS due to differences in resources, regulatory bodies, and the configurations of healthcare systems [73]. Financial factors often influence treatment decisions, sometimes leading to cost‐driven choices or non‐adherence. This impact is greater in market‐based systems like those in the USA [41], China [40], and Germany [30] compared to systems like the UKs, where care is free at the point of access. However, some pwALS may choose cheaper treatment options due to insufficient information provided by health care professionals (HCPs) regarding the availability of patient assistance programs and other mechanisms to reduce out‐of‐pocket drug costs. Increased access to this information could in turn broaden treatment choices. Similarly, differences in understanding of treatment implications can influence preferences. For example, while HCPs understand that introducing a PEG doesn't necessarily prevent pleasure from oral intake of food, some patients still report “wanting to eat independently, no matter how difficult” as a reason to avoid having a PEG [25]. This difference may stem from insufficient support for patients in understanding the consequences of their treatment choices.

### 4.1 Limitations

The review lacks comparative data of preferences within treatment and intervention types, limiting the reliability of findings. Some studies had biased sampling, focusing on participants with similar views e.g., all who accepted or declined an intervention before a user experience assessment [25, 29, 48]. This excludes views from differing perspectives, potentially reducing the generalisability of the results.

The preferences of pwALS reported in this review reflect the context of treatment at the time of data collection. Given the rapid evolution of ALS interventions, some preferences may no longer reflect current treatment realities. For example, while intravenous administration was a key reason for edaravone discontinuation, the more recent approval of an oral formulation may invalidate this. Similarly, PB‐TURSO discontinuation due to gastrointestinal side effects and taste is no longer applicable as the drug has been withdrawn from the US and Canadian markets and was never approved in Europe. However, these results still emphasize the preference of pwALS for less invasive modes of delivery and fewer disruptive side effects, and show consistencies that can be applied across different treatments.

### 4.2 Future Directions

The themes identified in this review can inform the attributes of discrete choice experiments (DCEs) to be used in the development of future preference‐based measures. Systematically varying the levels of these attributes in a DCE elicits quantitative measures of preferences. This approach enables a deeper understanding of the relative importances of different treatment characteristics and the trade‐offs pwALS are willing to make. Importantly, this can inform health care policy of the specific values of pwALS to optimize resource allocation and tailor clinical practice.

## 5 Conclusions

This systematic review across six intervention categories—drug treatments, nutritional support, special equipment, psychosocial support, exercise programmes, and digital health—revealed the key factors influencing preference decisions as: ease of use, accessibility, making life easier, autonomy, and safety/reliability. While not every theme emerged in every category, reflecting the distinct goals of different interventions (e.g., symptom management vs. disease progression), the consistent emergence of these themes across varied treatment types suggests fundamental, underlying values in ALS care that transcend specific treatment goals and modalities. These findings provide researchers, clinicians, and policymakers with evidence to inform practical, patient‐centered decisions regarding ALS treatments and interventions. Integrating patient preferences in clinical practice promotes patient‐centered care, which increases patient satisfaction and treatment effectiveness [74].

## Author Contributions

**A. Clift:** investigation, writing – original draft, methodology, writing – review and editing, formal analysis. **D. Rowen:** conceptualization, funding acquisition, investigation, methodology, formal analysis, supervision, writing – review and editing. **L. Knox:** investigation, methodology, supervision, formal analysis, writing – review and editing. **A. W. Griffiths:** investigation, methodology, supervision, formal analysis, writing – review and editing. **C. J. McDermott:** conceptualization, investigation, funding acquisition, methodology, supervision, writing – review and editing.

## Ethics Statement

We confirm that we have read the Journal's position on issues involved in ethical publication and affirm that this report is consistent with those guidelines.

## Conflicts of Interest

The authors declare no conflicts of interest.

## Data Availability

Data sharing is not applicable to this article as no new data were created or analyzed in this study.

## Appendix

**TABLE A1:** Inclusion and exclusion criteria.

Inclusion	Exclusion
Patients: Studies of adults (18+) with a diagnosis of Amyotrophic Lateral Sclerosis (ALS), Primary Lateral Sclerosis (PLS) and Progressive Muscular Atrophy (PMA) Studies: Published in English language since 2011. Outcomes: Studies reporting on preferences or experiences with MND/ALS treatments or care services. No restrictions were placed on the study design. This could include direct measurement of utilities for treatment outcomes, surveys regarding what factors are important to treatment decisions, and studies that rank importance of considerations. Experimental, quasi‐experimental and observational research studies, including qualitative and mixed methods study.	Abstracts or conference abstracts to which no associated full text is available. Studies reporting on family members or carers of people with MND only Studies published before 2011. Studies that do not assess relevant outcomes.

**TABLE A2:** Characteristics of the included studies and preference results.

Intervention	Author, year	Name of intervention	Location (s)	Type of study	Preference assessment	First order participant quotes/primary data	Second order codes	Preference theme
Drug treatment	Ludolf et al. 2021 [37]	Riluzole	Germany, Italy, Spain, France	Quantitative	Patient Preference Survey (PPS)	Low risk of choking and underdosing. Easier mode of delivery	Low risk Ease of administration	Safety/Reliability Ease of Use
	Seo et al., 2023 [38]	Intrathecal delivery methods, Implanted drug delivery device vs. lumbar puncture	USA, Germany, Italy, Spain, France, UK	Quantitative	Discrete choice experiment	The risk of device failure. Shorter overall durations and less frequent administration	Low risk Less disruptive administration	Safety/Reliability Ease of Use
	Lunette et al., 2020 [33]	Edaravone	Italy	Quantitative	Observational, real‐world assessment	22 patients voluntarily suspended from the burden of the duration and route of administration.	Shorter duration Easier route of administration	Ease of Use
	Quinn et al., 2024 [39]	phenylbutyrate‐taurursodiol (PB‐TURSO)	USA	Quantitative	Patient report recorded by carers	Main reasons for not taking were discomfort from the gastrointestinal side effects 17/29 (58.6%) and from the taste of the drug 8/29 (29.6%).	Side effects Taste	Ease of Use
	Meyer et al., 2019 [34]	Tetrahydrocannabinol (THC) and cannabidiol (CBD)	Germany	Quantitative	Net Promoter Score (NPS), Treatment satisfaction questionnaire(TSQM‐9)	10/32 found it difficult to use. 5/32 found it inconvenient or very inconvenient.	Ease of Use	Ease of Use
	Jia et al., 2024 [40]	Traditional chinese medicine (TCM)	China	Mixed Methods	Questionnaire then Interviews	“At least it doesn't have so many side effects” “Riluzole is too expensive. If it is cheaper, I would try it”	Side effects Cost	Ease of use Accessibility
	Zubair et al., 2024 [41]	Methylcobalamin (vitamin B12)	USA	Qualitative	Semi structured Interviews	“It would be good to have a standard syringe, because otherwise, you have to learn every time.” “The cost poses a barrier	Easier administration Cost	Ease of Use Accessibility
Nutrition	Labra et al., 2020 [42]	PEG	Australia	Mixed methods	Interviews and analysis of physical function	“I am thinking that it will help me because you can still go out” “can use PEG to keep up my nutrition”	Maintaining independence Nutritional benefit	Autonomy Making life easier
	Guillemin et al., 2022 [45]	Nutrition interventions	Canada	Mixed methods	Survey	“Difficult to use knives and other utensils” 6 (60%) had weight loss concerns.	Ease in food preparation	Making life easier
	Starvoulakis et al., 2013 [43]	PEG	UK	Qualitative	Semi structured interviews 3 months post PEG	“Swallowing was becoming more difficult, “That's when she said Let's get it done”.	Prolonged, tiring and effortful meals Choking and aspiration	Making life easier
	van Eenennaam, et al., 2022 [44]	PEG	Netherlands	Qualitative	Semi structured Interviews	“I also wanted to continue to eat independently, no matter how difficult it was”.	Feeling of control	Autonomy
	Hogden, Labra and Power, 2024 [46]	PEG	Australia	Qualitative	Semi structured interviews	“My main reason for getting the PEG was my swallowing”	Optimizing quality of life	Making life easier
Special equipment	Greenaway et al., 2015 [24]	PEG and NIV	UK	Qualitative	Semi structured Interviews	“As far as I'm concerned it's my life, what's left” “Yeh, it's the worry that it'll get infected”	Perceptions of choice and control Aspects of fear	Autonomy Safety and Reliability
	Martin et al., 2014 [25]	PEG and NIV	UK	Quantitative	Interviews and beliefs about medicine questionnaire (BMQ)	More pleasure in eating were more likely to have refused an intervention	Control over illness	Autonomy
	Ando et al., 2015 [48]	NIV	UK	Qualitative	Semi structured interviews	“Wouldn't want it to be all of the time and not have any life” “claustrophobia – I just don't want it.”	Preservation of the self Negative perceptions of mask	Autonomy Safety and Reliability
	Baxter et al., 2013 [47]	NIV	UK	Qualitative	Semi structured interviews in first month of NIV	““I can get the mask on…but I can't, it's the clips.” “leaks just from the mask” “that mask on my face”	Ease of set up Mask dysfunction Negative perceptions of the mask.	Ease of Use Safety and Reliability
	Siewers, Holmoy & Frich, 2013 [26]	Cough assist	Canada	Qualitative	Interviews	“And it's not very complicated either. I think that will be important”. “So knowing that it is there	Ease of Use Comfort in reliability	Ease of Use Safety and Reliability
	Huggins, Wren & Gruis, 2011 [27]	Brain computer interface	USA	Quantitative	Survey using 1 to 10 scale	Accuracy followed by simplicity as most important. Appearance was least important	Accuracy	Safety and Reliability
	Geronimo et al., 2015 [49]	Brain computer interface	USA	Quantitative	10 min demonstration then survey	38/42 (90.5%) of ppts would like 90% accuracy and 50% required at least 80%.	Accuracy	Safety and Reliability
	Eicher et al., 2019 [50]	Assistive technology	Germany	Qualitative	Semi structured interviews	“It would be great if could also disburden somebody” Patient was afraid…it might fall off the ceiling and hit him	Maintain and retrieve autonomy Safety	Autonomy Safety and Reliability
	Creemers et al., 2014 [51]	Assistive devices and home adaptations	Netherlands	Mixed methods	Survey	Long duration of application process, paperwork, knowledge and errors by supplier	Delays in procurement process	Ease of Use
	Riera‐Punet et al., 2019 [28]	Appliance for oral self biting	Spain	Quantitative	Intervention for 3 months then questionnaire	The main reason for not using it was discomfort. Difficulty making an impression to make the mold due to MND	Comfort Physical barriers	Ease of Use Accessibility
	Spears et al., 2024 [35]	Neck collar	UK	Quantitative	Online Survey	“Too uncomfortable” (51%) and “too restricting” (44%) led to users not wearing. (96%) want the collar to be well fitting.	Comfort	Ease of Use
	Mackenzie et al., 2016 [52]	Communication aid technology	Australia	Mixed methods	Survey with closed and open questions	“Since my hands ceased being able to move I have been isolated from this”. “MND stops a person being independent, but with a computer (she) was able to communicate”.	Lack of physical ability Reduce isolation	Accessibility Making life easier
	Peters, O'Brien & Fried‐Oken, 2022 [29]	Augmentative and alternative communication devices (ACC)	USA	Quantitative	Questionnaire	High cost due to insurance co‐payments. Concerns about using the device and it not working well	Cost Risk of device failure	Accessibility Safety and Reliability
	Caligari et al., 2013 [53]	Eye tracking communication aids	Italy	Quantitative	Quebec User Evaluation of Satisfaction with Assistive Technology (QUEST 2.0)	Side effects of bloodshot eyes from use. Regaining autonomy in personal life choices	Side effects Regained autonomy	Ease of Use Autonomy
	Cave and Bloch, 2020 [54]	Voice banking	UK	Qualitative	Interviews	“The voice helps me retain something of me.” “Its about control”	Preserving identity Control	Autonomy
	Spittel et al., 2024 [30]	Robotic arm	Germany	Quantitative	User experience survey	Issues were, the unsuitability of the device 16/53 (30.2%) followed by rejection by the health care insurance 15/53 (28.3%)	Adaptability to MND Cost	Accessibility
Psychosocial support	Weeks et al., 2019 [56]	Psychological interventions	UK	Qualitative	Individual semi structured interviews then discussion groups	“having a therapist coming home would be very useful” “can't physically communicate	Adaptability to MND.	Accessibility
	Pinto et al., 2023 [57]	Digital mental health tools	UK	Qualitative	Think aloud interviews then intervention for 6 weeks then in depth interviews.	More appropriate I think to people with motor neuron” Taking some control over what is happening to them.	Realistic advice for MND Regaining control	Accessibility Autonomy
	Hardy, Castle & Jackson, 2022 [31]	Psychiatric care	USA	Quantitative	Survey with 5 point Likert scale	Value the availability of psychiatric services even when have no symptoms	Accessibility	Accessibility
	Marconi et al., 2016 [58]	Meditation	Italy	Qualitative	Weekly meditation for 8 weeks followed by interviews	It was difficult for us to organize transportation” “wasn't easy to do exercises at home”	Transportation Continuity	Accessibility
	Bentley et al., 2014 [32]	Dignity therapy	Australia	Quantitative	Dignity therapy followed by feedback questionnaire	Positive improvements in continuity of self and role preservation.	Preservation of identity	Autonomy
	Sommers‐Spijkerman et al., 2024 [59]	Self compassion intervention	Netherlands	Mixed methods	Survey and Interviews	“Thanks to this app I think ‘What positive things happened today?’” Participants struggled to integrate the intervention into everyday life 10/13 (63%).	Positive shift in attention Continuity	Making life easier Accessibility
	Wu et al., 2022 [55]	Respite care	Canada	Qualitative	Interviews	Readjusting and them re‐learning” “wouldn't want them to bathe her”	Consistency Privacy	Ease of Use Autonomy
Exercise programme	Maier et al., 2022 [60]	Motor assisted movement exercises	Germany	Quantitative	5 and 11 point Likert scale, Net Promoter Score (NPS) and interviews	Key impacts include a sense of achievement (67%), reduced immobility (61%), and improved well‐being (55%). Symptom relief with reduced limb rigidity (63%) and muscle stiffness (52%).	Improved quality of life Symptom relief	Making life easier
Digital health	Fidelix et al., 2023 [36]	Telehealth for multidisciplinary care	Brazil	Quantitative	Teleconsultation followed by questionnaire	4/36 (11%) patients disagreed that it was easy to set up convenience of receiving assistance and maintenance of multidisciplinary care at home.	Ease of set up Continuity of care	Ease of Use Accessibility
	Helleman et al., 2020 [62]	Telehealth for home monitoring	Netherlands	Mixed methods	Survey then interviews	““Logging in is difficult with the digital ID” “You don't feel you are going for no good reason” positive about receiving personalized feedback and information	Log in issues Reducing the unnecessary personalisation	Ease of Use Making life easier Accessibility
	James et al., 2019 [63]	Videoconferencing and email	Australia	Mixed methods	Survey then interviews	“it wouldn't take so much energy out of my life and I think my day would be a bit better”	Reducing the unnecessary	Making life easier
	Hobson et al., 2017 [64]	Every day digital health technology e.g., ipads, laptops	UK	Mixed methods	Questionnaire then interviews	“Anything that makes life's journey, when necessary, better” 9/12 said they would use technology with the appropriate equipment and training.	Making life easier Accessibility	Making life easier Accessibility
	Ando et al., 2021 [65]	Telehealth for home monitoring	UK	Qualitative	Telemonitoring for NIV for 24 weeks then interviews	“It's save a lot of money as well as you know because I won't need to keep going to hospital” “The keyboard is that frustrating. I just couldn't be bothered trying to get it to work because it wouldn't.”	Reducing the unnecessary Technical challenges	Making life easier Safety and reliability
	Helleman et al., 2022 [66]	Telehealth for home monitoring and clinical trials	Netherlands, UK, Australia	Quantitative	Survey	86.1% were willing to record at least monthly and 60.7% at least weekly. Concerns with privacy, data security and data being sold to third parties.	Low cognitive burden Technical reliability	Accessibility Safety and reliability
	Tattersall et al., 2022 [67]	Remote respiratory assessment	Ireland	Mixed methods	At home assessments via webcam then a survey	Difficulty from mobility and speech issues Reducing the number of clinic visits was valuable in 24/25 of the pwALS	Adaptability to MND Reducing appointments	Accessibility Making life easier
	Beswick et al., 2024 [61]	ActiGraph GT9X wearable device for movement evaluation	Scotland	Quantitative	Questionnaire	Difficulty putting on the devices and faulty straps. Large size of the device affected their comfort Positive response that it meant less clinical appointments.	Physical ability, Comfort Reducing appointments	Ease of use Accessibility Making life easier

**TABLE A3:** Quality assessment for the systematic review treatment preferences in people with ALS using the mixed methods appraisal tool (MMAT) criteria for qualitative (1.1–1.5) quantitative descriptive (4.1–4.5) and mixed methods (5.1.‐5.5) studies.

Author, date	Treatment	Criteria from the mixed method appraisal tool														
		1.1	1.2	1.3	1.4	1.5	4.1	4.2	4.3	4.4	4.5	5.1	5.2	5.3	5.4	5.5
		Qualitative					Quantitative					Mixed methods				
Ludolf et al., 2023	Drug treatment															
Lunetta et al., 2020	Drug treatment															
Meyer et al., 2019	Drug treatment															
Seo et al.2023	Drug treatment															
Jia et al., 2024	Drug treatment															
Zubair et al., 2024	Drug treatment															
Quinn et al., 2024	Drug treatment															
Greenaway et al., 2015	Nutrition/respiration															
Martin et al., 2014	Nutrition/respiration															
Ando et al., 2014	Respiratory support															
Baxter et al., 2013	Respiratory support															
Siewers, Holmoy & Frich, 2013	Respiratory support															
Labra et al., 2020	Nutritional support															
Guillemin et al., 2022	Nutritional support															
Starvoulakis et al., 2013	Nutritional support															
Eenennaam, et al., 2022	Nutritional support															
Hogden, Labra and Power, 2024	Nutritional support															
Huggins, Wren & Gruis, 2011	Brain computer interface															
Geronimo et al., 2015	Brain computer interface															
Weeks et al., 2019	Psychosocial support															
Pinto et al., 2023	Psychosocial support															
Marconi et al., 2016	Psychosocial support															
Bentley et al., 2014	Psychosocial support															
Hardy, Castle & Jackson, 2022	Psychosocial support															
Sommers‐Spijkerman et al., 2024	Psychosocial support															
Wu et al., 2022	Psychosocial support															
Eicher et al., 2019	Supporting daily living															
Creemers et al., 2014	Supporting daily living															
Riera‐Punet et al., 2019	Supporting daily living															
Spears et al., 2024	Supporting daily living															
Spittel et al., 2024	Supporting daily living															
Mackenzie et al., 2016	Communication aids															
Peters, O'Brien & Fried‐Oken, 2022	Communication aids															
Cave & Bloch 2020	Communication aids															
Caligari et al., 2013	Communication aids															
Fidelix et al., 2023	Digital health															
Helleman et al., 2020	Digital health															
James et al., 2019	Digital health															
Hobson et al., 2017	Digital health															
Ando et al., 2021	Digital health															
Helleman et al., 2022	Digital health															
Tattersall et al., 2022	Digital health															
Beswick et al., 2024	Digital health															
Maier et al., 2022	Excersise programme															

*Note:* Achieved (Black), not achieved (light gray), inconclusive (dark gray).
